# Long-term oncologic outcomes and prognostic factors related to recurrences in pathologic Stage I/II early oral tongue cancer

**DOI:** 10.3389/fsurg.2025.1534274

**Published:** 2025-05-21

**Authors:** Jin Hye Kwak, Yong Bae Ji, Chang Myeon Song, Young-Jun Lee, Hae Jin Park, Kyung Tae

**Affiliations:** ^1^ Department of Otolaryngology-Head and Neck Surgery, College of Medicine, Hanyang University, Seoul, Republic of Korea; ^2^ Department of Radiology, College of Medicine, Hanyang University, Seoul, Republic of Korea; ^3^ Department of Radiation Oncology, College of Medicine, Hanyang University, Seoul, Republic of Korea

**Keywords:** tongue cancer, early stage, early recurrence, prognosis, disease-free survival, overall survival

## Abstract

**Objectives:**

This study aimed to evaluate long-term treatment outcomes and identify prognostic factors affecting survival and recurrence of Stage I/II tongue cancer, with a specific emphasis on early recurrence.

**Methods:**

We retrospectively studied 65 patients with pathologic Stage I/II oral tongue squamous cell carcinoma (OTSCC), who underwent definitive surgical treatment, with or without postoperative adjuvant therapy, from 1997 to 2022.

**Results:**

Thirteen (20%) cases experienced recurrence. The 2-, 5-, and 10-year overall survival rates were 91.7%, 88.0%, and 81.5%, respectively. Univariate Cox analyses showed that recurrence and overall survival were significantly associated with Stage II, depth of invasion (DOI) > 5 mm, lymphovascular invasion (LVI), and perineural invasion (PNI). Multivariate Cox analysis identified LVI as an independent predictor of recurrence and PNI as an independent predictor of survival. Second primary tumors in the head and neck region occurred in 10.8%, and both recurrence and second primary tumors significantly decreased survival in univariate and multivariate analyses. Early recurrence (within 6 months post-surgery) occurred in 3 patients (23.1% of recurrences), with no significant predictors identified by Cox analysis.

**Conclusions:**

Recurrence and survival of early OTSCC are associated with Stage II disease, high DOI, PNI, and LVI in univariate analysis. Also, in patients with early OTSCC, prevention and proper treatment of second primary tumors and recurrence are crucial for improving overall survival.

## Introduction

Tongue cancer is the most prevalent type of oral cavity cancer worldwide (1). It exhibits an incidence rate of 3.6 per 100,000 and a mortality rate of 0.7 per 100,000 in the United States (2). Despite an overall decline in the incidence of oral cancer, there has been a consistent upward trend in the incidence of tongue cancer over the past four decades (2, 3). Importantly, its occurrence has shifted toward younger (<45 years of age) and female patients (3). Notably, individuals who have never smoked or consumed alcohol have become more prevalent (4).

According to the SEER 22 database, the 5-year relative survival of tongue cancer from 2013 to 2019 was 69.7% (2). It was reported that 29% were local diseases, 54% had regional lymph node metastasis, and 12% had distant metastasis (2). Survival rates substantially decreased with increasing stage severity. The 5-year overall survival rate drops from 75% to 50% with lymph node metastasis and 30% with extracapsular spread (5). Early detection and intervention may improve prognosis.

Oral tongue squamous cell carcinoma (OTSCC) is the most frequent subtype of tongue cancer and is notorious for its high recurrence rate and poor prognosis (1, 4, 6). Even with aggressive treatment of the primary tumor and neck metastasis, recurrence is common. Recurrence rates range from 18% to 76%, with over 80% occurring within 24 months after treatment (7, 8). The median time to recurrence is 7.5 months (7, 8).

The determinants of OTSCC include smoking/chewing tobacco, alcohol, and chronic trauma (9, 10). Local-regional recurrence has also been linked to a positive surgical margin, poor histopathological grading, high depth of invasion (DOI), lymphovascular invasion (LVI), perineural invasion (PNI), and lymph node metastasis (11–15). Although several prognostic factors have been identified, predicting the recurrence of tongue cancer is challenging. Unexpected recurrences can occur even in early-stage cancer. Factors related to early recurrences of OTSCC in early-stage cancer remain unclear. Therefore, this study aimed to investigate the long-term treatment outcomes of early OTSCC and the factors associated with recurrence, especially early recurrence.

## Materials and methods

### Patients and data collection

This retrospective study included 65 patients diagnosed with pathologic Stage I/II OTSCC, who underwent definitive surgical treatment between 1997 and 2022. The inclusion criteria were patients with biopsy-proven pathologic Stage I/II OTSCC who underwent primary surgical resection of the tongue lesion with or without neck dissection and postoperative adjuvant therapy. Exclusion criteria included patients with pathological findings different from squamous cell carcinoma, patients with a different subsite of oral cancer, cases with occult positive node at the final pathologic report, those undergoing curative radiation, concurrent chemoradiation therapy (CCRT), or palliative treatment, and patients lacking clinical data.

The Institutional Review Board (IRB) of Hanyang University Hospital (IRB number 2022-11-021) approved the study. In conducting this study, we followed the principles outlined in the Declaration of Helsinki and good clinical practice guidelines.

Demographic and clinical data, such as age, sex, smoking and alcohol consumption history, histopathological features, and treatment modality, were obtained from medical records. Pathological staging and classification were based on the eighth edition of the American Joint Committee on Cancer's (AJCC 8th) staging manual. DOI was primarily obtained from pathology reports. However, pathologists occasionally used alternative terms such as “tumor thickness” or “tumor invasion thickness”. Although, by strict definition, tumor thickness (vertical distance from the tumor's most superficial surface to its deepest point of invasion) and DOI (vertical distance from the adjacent normal mucosal basement membrane to the deepest point of invasion) are different, cross-validation through retrospective comparison with magnetic resonance (MR) imaging confirmed for the cases of our patients that these terms corresponded closely to DOI without significant discrepancies affecting the T classification. Radiological DOI was measured on MR images as the perpendicular distance from a reference line drawn at the junction of the tumor with the adjacent normal mucosal surface down to the tumor's deepest invasive point. Surgical margins greater than 2 mm were considered negative, margins of 2 mm or less were categorized as close, and margins with tumor involvement were defined as positive. The presence of PNI and LVI were obtained from pathology reports, which were available for 63 patients.

All patients underwent preoperative imaging study, such as computed tomography (CT), MR, ultrasonography, and positron emission tomography (PET) to evaluate the clinical staging of the primary tumor and lymph node status. If suspicious lymph nodes were identified on imaging studies, fine needle aspiration cytology was conducted.

In our institute, the primary treatment principle for OTSCC was primary resection of tongue cancer, with or without neck dissection. For early-stage OTSCC, transoral surgical resection of the primary tumor was performed. For more advanced cases, pull-through, mandibulo-lingual release, and, in rare instances, mandibulotomy were necessary. Prophylactic selective neck dissection (SND) was performed in most cases except in cases with superficial T1 cancers, where the DOI was assessed as 1–2 mm based on preoperative imaging, primarily MR. The extent of prophylactic SND included levels I-III in most cases or levels I–IV in other cases based on the surgeons' preference and disease status.

A multidisciplinary team meeting with the patients was held to decide on further treatments. Adjuvant radiotherapy (RT) or chemoradiation therapy was administered 4–6 weeks after surgery to selected patients who were identified as having high-risk features such as close or positive surgical margins, high DOI, PNI, and LVI.

To detect recurrence, physical examinations, neck CT, and ultrasonography were conducted at 3–6-month intervals for 2 years postoperatively, then every 12 months for up to 5 years, and then annually or biannually. Structural recurrence was defined as the emergence of new abnormal structural lesions evident in imaging studies and pathologically confirmed via biopsy or fine-needle aspiration cytology. Early recurrence was defined as occurring within 6 months after surgery.

### Statistical analysis

Statistical analyses were conducted using SPSS version 28.0 software (IBM SPSS Statistics, Somers, NY, USA). The chi-square test or Fisher's exact test was used to compare categorical variables, and the *t*-test was used to compare continuous variables. Kaplan–Meier curves were generated to estimate overall survival (OS) and disease-free survival (DFS), and differences between survival curves were tested using the log-rank test. The Cox proportional hazards models were used for univariate and multivariate analyses to identify factors related to recurrence and survival. Variables with a *p* < 0.05 in univariate analyses were included in the multivariate Cox regression models using backward stepwise selection based on the likelihood ratio. A *p*-value of less than 0.05 was considered statistically significant.

## Results

### Clinicopathologic characteristics and surgical outcomes

The demographics and clinical characteristics of patients with pathologic Stage I/II OTSCC are presented in Table 1. Of the 65 patients, 52.3% were male, and 47.7% were female. The mean age was 58.4 ± 15.6 years. Smokers comprised 43.1%, while 41.5% had a history of alcohol consumption. Stage I disease was detected in 52.3% and Stage II in 47.7%. None of the patients had lymph node metastasis. The mean DOI was 5.2 ± 2.8 mm. DOI > 5 mm occurred in 43.1%. Surgical margins were negative in 89.2% of cases, 6.2% had close margins, and 4.6% had positive surgical margins. Histologically, most tumors were well-differentiated (62.3%). LVI was present in 7.9%, and PNI in 11.1% of the patients.

**Table 1:** Demographics and clinicopathologic characteristics of patients with pathologic Stage I/II oral tongue cancer (*N* = 65).

Characteristics	*N* (%)
Sex	
Male	34 (52.3%)
Female	31 (47.7%)
Age (mean, year)	58.4 ± 15.6
Smoking	
Non-smoker	37 (56.9%)
Smoker (former, current)	28 (43.1%)
Alcohol drinking	
Non-drinker	38 (58.5%)
Drinker (social, heavy drinker)	27 (41.5%)
Surgical approach	
Transoral	64 (98.5%)
Pull-through	1 (1.5%)
Extent of glossectomy	
Partial glossectomy	50 (79.6%)
Hemiglossectomy	14 (21.5%)
Subtotal glossectomy	1 (1.5%)
Selective neck dissection	
No	13 (20.0%)
Yes	52 (80.0%)
Selective neck dissection (level I–III)	46 (70.8%)
Selective neck dissection (level I–IV)	6 (9.2%)
Stage (AJCC 8th)	
I	34 (52.3%)
II	31 (47.7%)
T classification	
1	34 (52.3%)
2	31 (47.7%)
N classification	
0	65 (100%)
Depth of invasion (mean, mm)	5.2 ± 2.8
≤5 mm	37 (56.9%)
>5 mm	28 (43.1%)
Surgical margin	
Negative (>2 mm)	58 (89.2%)
Close (≤2 mm)	4 (6.2%)
Positive	3 (4.6%)
Histologic grade (*N* = 61)	
Well-differentiated	38 (62.3%)
Moderately differentiated	21 (34.4%)
Poorly differentiated	2 (3.3%)
Lymphovascular invasion (*N* = 63)	5 (7.9%)
Perineural invasion (*N* = 63)	7 (11.1%)
Adjuvant treatment	
None	57 (87.7%)
Radiotherapy	7 (10.8%)
Concurrent chemoradiation	1 (1.5%)
Recurrence	
No	52 (80.0%)
Yes	13 (20.0%)
Local	4 (6.2%)
Regional	4 (6.2%)
Loco-regional	4 (6.2%)
Distant	1 (1.5%)
Second/Multiple primary cancer	7 (10.8%)
Hypopharynx (pyriform sinus)	2
Oral cavity (alveoli, buccal, tongue)	3
Oropharynx (tonsil)	1
Oral cavity (buccal) and oropharynx (tonsil)	1
Follow-up (mean, months)	76.9 ± 56.8

The majority (98.5%) of patients underwent transoral glossectomy. Partial glossectomy was performed in 79.6%, hemiglossectomy in 21.5%, and subtotal glossectomy with removal of approximately two-thirds of the tongue in 1.5%. Neck dissection was performed in 80% of patients (SND levels I-III in 70.8% and SND levels I-IV in 9.2%). Postoperative adjuvant treatment was administered to 8 patients (12.3%); RT to 7 patients (10.8%) and CCRT in one patient (1.5%) with positive surgical margin and DOI 9 mm. Postoperative RT doses ranged from 6,000 to 7,000 cGy (predominantly 6,300 cGy) and were delivered over 28 to 35 fractions. Some cases refused adjuvant therapy although it was indicated in the criteria and opted for close follow-up.

### Factors related to recurrence and survival

Recurrence occurred in 13 (20%) patients, with a mean follow-up of 76.9 months. The mean time to recurrence was 40.7 months, with the earliest recurrence detected at 3.4 months after surgery. Most recurrences occurred loco-regionally; four patients experienced local recurrence, four had regional recurrence, and four had both local and regional recurrences. Distant metastasis was observed in one patient.

Among the eight patients with regional recurrence in the neck lymph nodes, two had not undergone neck dissection during primary treatment, while the remaining six had undergone SND of levels I-III. Although including patients without primary SND could introduce bias due to potential occult metastasis, their recurrence patterns and pathological results suggest that recurrences were unlikely to stem from pre-existing occult nodal disease.

Among the 13 recurrences, 8 patients underwent salvage surgery with or without postoperative chemotherapy or radiotherapy. The remaining 4 received palliative treatment (radiotherapy/chemotherapy) and 1 patient refused treatment (Supplementary Table S1).

Second primary cancer (located in the oral cavity, oropharynx, or hypopharynx) occurred in seven (10.8%) patients (Table 1).

According to the chi-square and Fisher's exact analyses, Stage II, LVI, and DOI > 5 mm were associated with recurrence (Table 2). The univariate Cox proportional hazards regression analysis identified Stage II disease [odds ratio (OR) of 4.04 and 95% confidence interval (CI) of 1.11–14.67], LVI [OR and 95% CI, 6.74 (1.77–25.68)], PNI [OR and 95% CI, 5.07 (1.32–19.43)], and DOI > 5 mm [OR and 95% CI, 3.40 (1.05–11.06)], as significant predictors for recurrence (*p* < 0.05) in Table 3. Variables significant in the univariate analysis were entered into the multivariate analysis to identify independent prognostic factors. LVI emerged as the only statistically significant independent predictor [OR and 95% CI, 5.06 (1.30–19.71)] for recurrence (Table 3).

**Table 2:** Clinicopathologic factors related to recurrence (*N* = 65).

Factors		Recurrence (−) (*N* = 52)	Recurrence (+) (*N* = 13)	*p*-value
Sex	Male	30	4	
	Female	22	9	0.08
Age (year)	<65	34	9	
	≥65	18	4	1.00
Smoking	Non-smoker	28	9	
	Smoker	24	4	0.32
Alcohol	Non-drinker	30	8	
	Drinker	22	5	0.80
Stage	I	31	3	
	II	21	10	0.02
Histologic grade (*n* = 61)	Well	32	6	
	Moderately/Poorly	17	6	0.34
Lymphovascular invasion (*n* = 63)	No	49	9	
	Yes	2	3	0.04
Perineural invasion (*n* = 63)	No	47	9	
	Yes	4	3	0.12
Depth of invasion	≤5 mm	33	4	
	>5 mm	19	9	0.03
Surgical margin	>2 mm	48	10	
	≤2 mm	4	3	0.14
Adjuvant treatment	No	46	11	
	Yes	6	2	0.66

**Table 3:** Cox proportional hazards regression model for recurrences.

Variables	Univariate		Multivariate	
	OR (95% CI)	*p*-value	OR (95% CI)	*p*-value
Sex (Female)	3.01 (0.92–9.85)	0.07		
Age (≥65)	0.90 (0.28–2.93)	0.86		
Smoking	0.58 (0.18–1.89)	0.37		
Alcohol drinking	0.75 (0.24–2.29)	0.61		
Stage (II)	4.04 (1.11–14.67)	0.03	3.28 (0.87–12.41)	0.08
Histologic grade (moderately/poorly)	1.75 (0.56–5.42)	0.33		
Lymphovascular invasion	6.74 (1.77–25.68)	0.005	5.06 (1.30–19.71)	0.02
Perineural invasion	5.07 (1.32–19.43)	0.02	3.67 (0.67–20.11)	0.13
DOI >5 mm	3.40 (1.05–11.06)	0.04	0.78 (0.09–6.82)	0.82
Surgical margin (≤2 mm)	2.82 (0.78–10.27)	0.12		

OR, odds ratio; CI, confidence interval; DOI, depth of invasion.

For overall survival, the univariate Cox analysis revealed that Stage II disease [OR and 95% CI, 10.38 (1.31–82.06)], LVI [OR and 95% CI, 7.56 (1.86–30.78)], PNI [OR and 95% CI, 6.27 (1.53–25.62)], DOI > 5 mm [OR and 95% CI, 12.93 (1.64–102.13)], recurrence [OR and 95% CI, 11.87 (3.05–46.28)], and the development of second or multiple primary cancers [OR and 95% CI, 3.93 (1.01–15.25)] were all significantly associated with decreased overall survival (Table 4). The multivariate analysis showed that recurrence [OR and 95% CI, 30.82 (3.82–248.75)], second primary cancers [OR and 95% CI, 8.29 (1.46–47.13)], and PNI [OR and 95% CI, 6.56 (1.09–39.01)] were identified as independent prognostic factors significantly associated with decreased overall survival (Table 4).

**Table 4:** Cox proportional hazards regression model for overall survival.

Variables	Univariate		Multivariate	
	OR (95% CI)	*p*-value	OR (95% CI)	*p*-value
Sex (Female)	3.01 (0.77–11.72)	0.11		
Age (≥65)	0.55 (0.12–2.62)	0.45		
Smoking	0.89 (0.25–3.16)	0.86		
Alcohol drinking	0.76 (0.21–2.69)	0.66		
Stage (II)	10.38 (1.31–82.06)	0.03	<0.01 (<0.01–1.83 × 10^142^)	0.96
Histologic grade (moderately/poorly)	1.66 (0.41–6.63)	0.48		
Lymphovascular invasion	7.56 (1.86–30.78)	0.005	1.01 (0.18–5.63)	0.99
Perineural invasion	6.27 (1.53–25.62)	0.01	6.56 (1.09–39.01)	0.04
DOI >5 mm	12.93 (1.64–102.13)	0.02	1.96 × 10^4^ (<0.01–1.02 × 10^150^)	0.95
Surgical margin (≤2 mm)	3.47 (0.89–13.42)	0.07		
Recurrence	11.87 (3.05–46.28)	<0.001	30.82 (3.82–248.75)	0.001
Second primary	3.93 (1.01–15.25)	0.048	8.29 (1.46–47.13)	0.02

OR, odds ratio; CI, confidence interval; DOI, depth of invasion.

Of the 13 patients with recurrence, three (23.1%) had early recurrences within 6 months after surgery, and all of them were female with Stage II disease, DOI > 5 mm, and PNI. According to Fisher's exact test (*p* = 0.001) (Supplementary Table S2), PNI was the factor associated with early recurrence. However, the Cox proportional hazards regression model for early recurrences did not detect any significant factors related to early recurrence (Supplementary Table S3).

When comparing early vs. late recurrences in patients with early OTSCC, the presence of PNI was the only statistically significant factor (*p* = 0.005) (Supplementary Table S4).

### Overall and disease-free survival analysis

**Figure 1:**
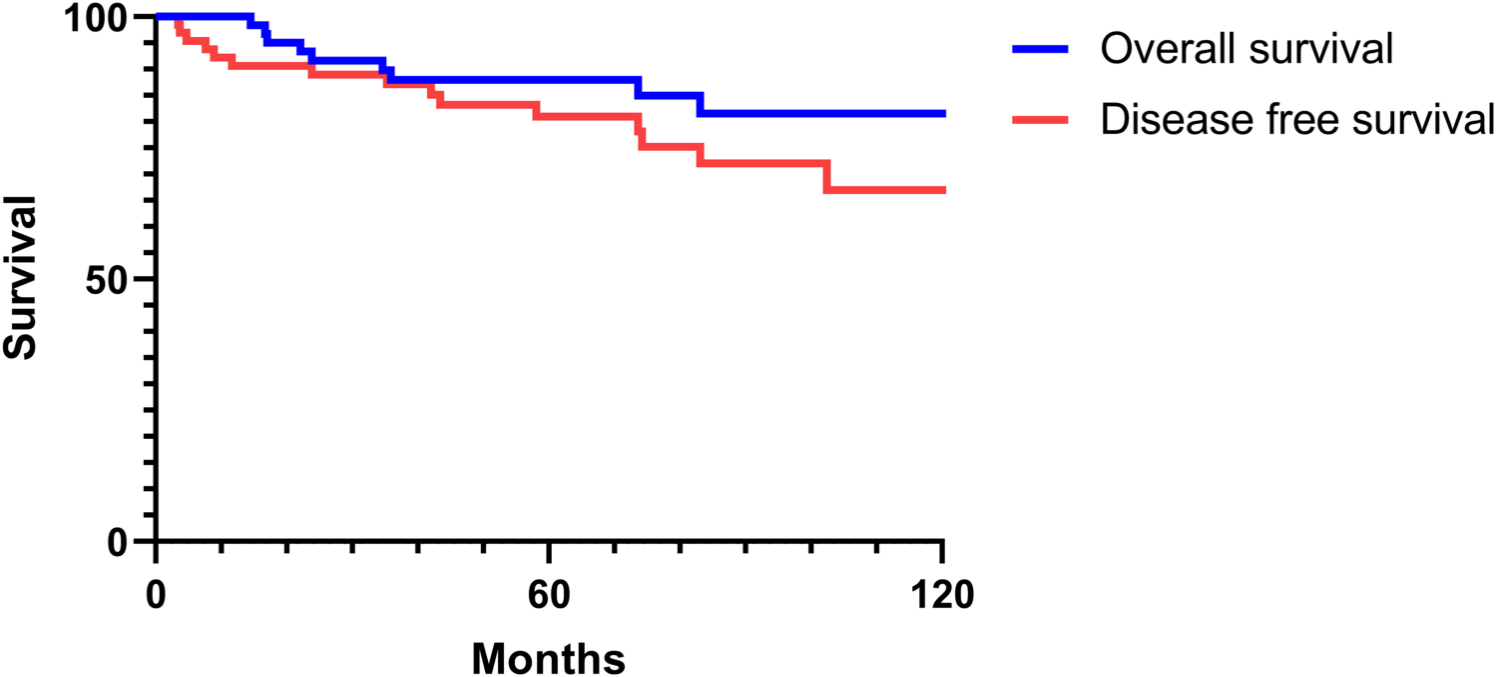
Overall and disease-free survival curves of Stage I/II oral tongue squamous cell carcinoma.

**Figure 2:**
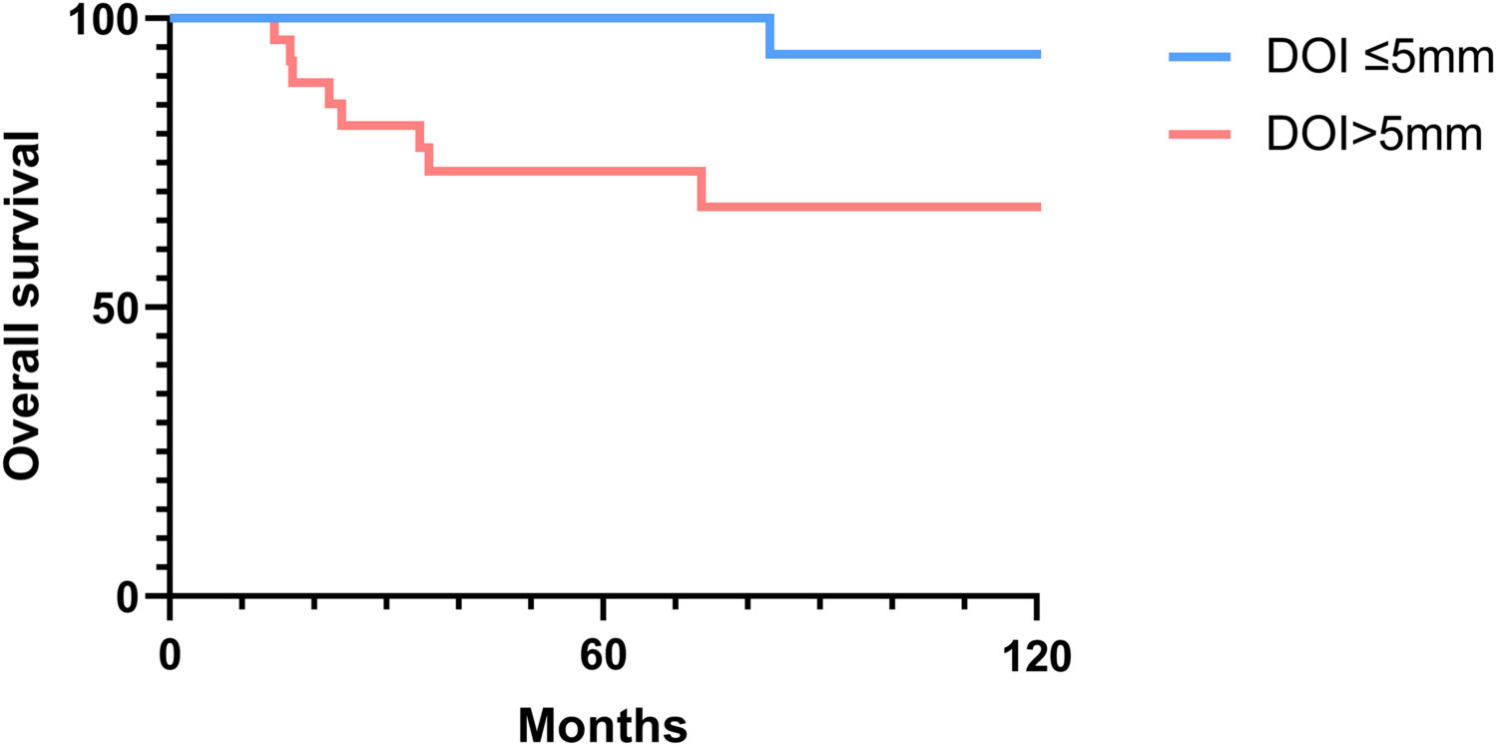
Overall survival curves showing significant differences according to the depth of invasion (DOI).

**Figure 3:**
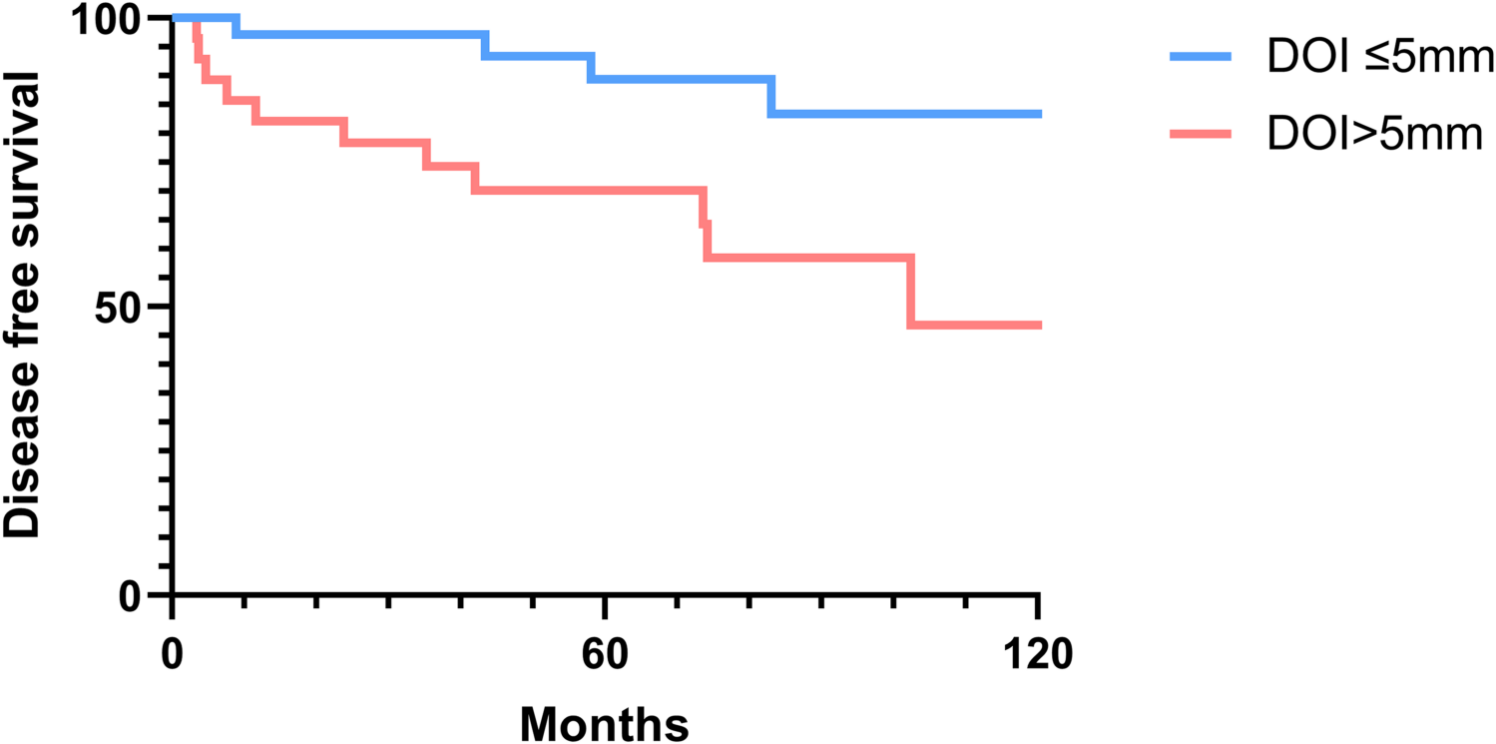
Disease-free survival curves showing significant differences according to the depth of invasion (DOI).

**Figure 4:**
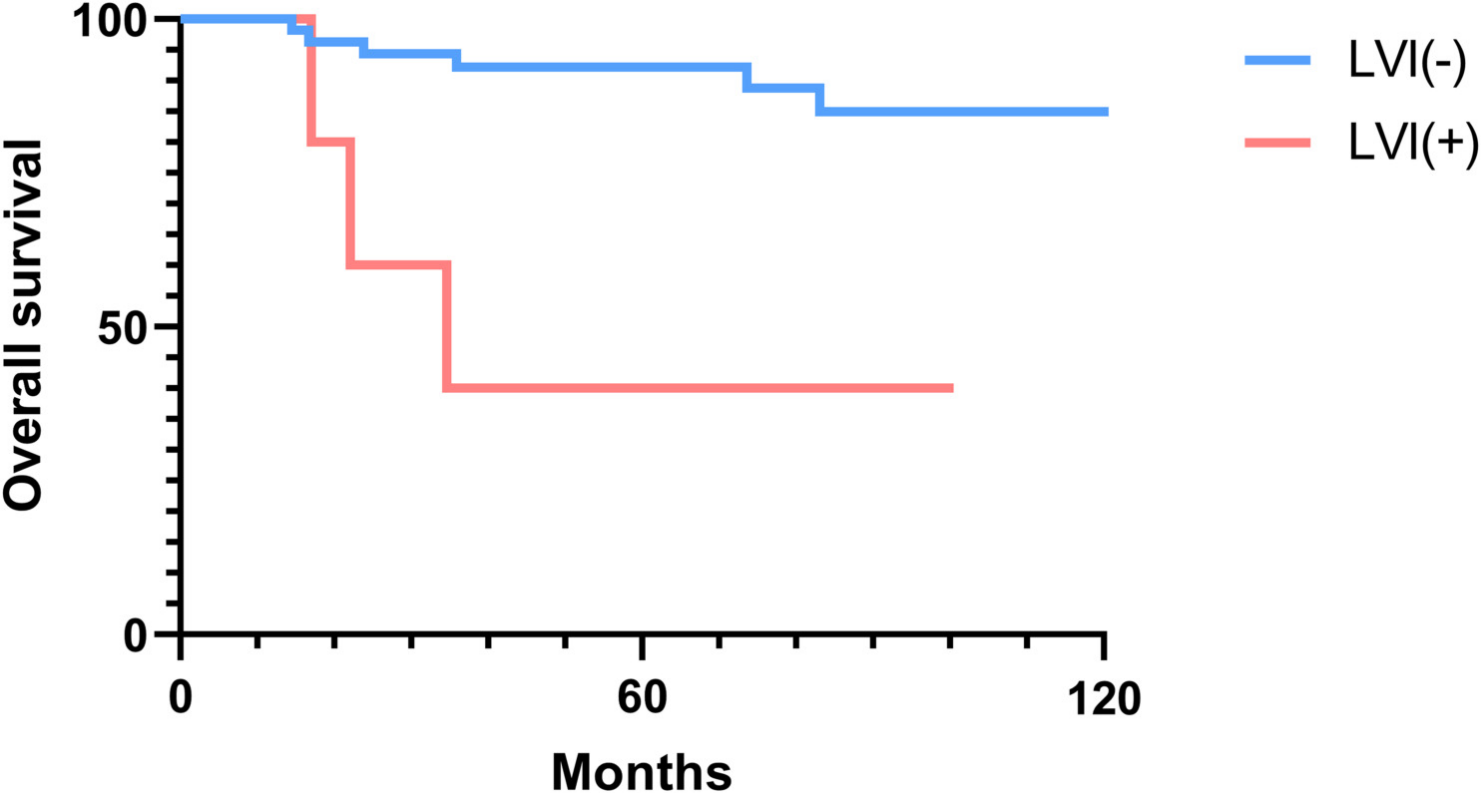
Overall survival curves showing significant differences according to lymphovascular invasion (LVI).

**Figure 5:**
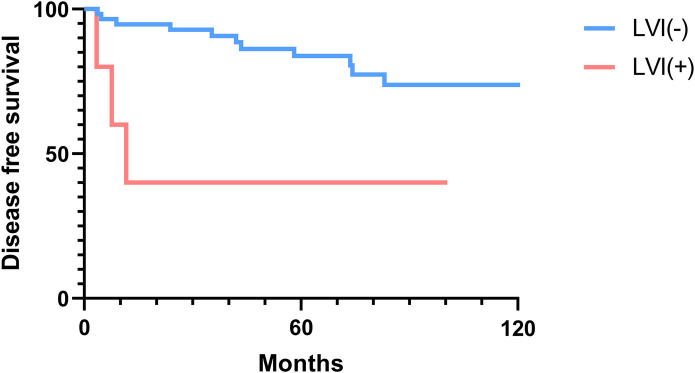
Disease-free survival curves showing significant differences according to lymphovascular invasion (LVI).

**Figure 6:**
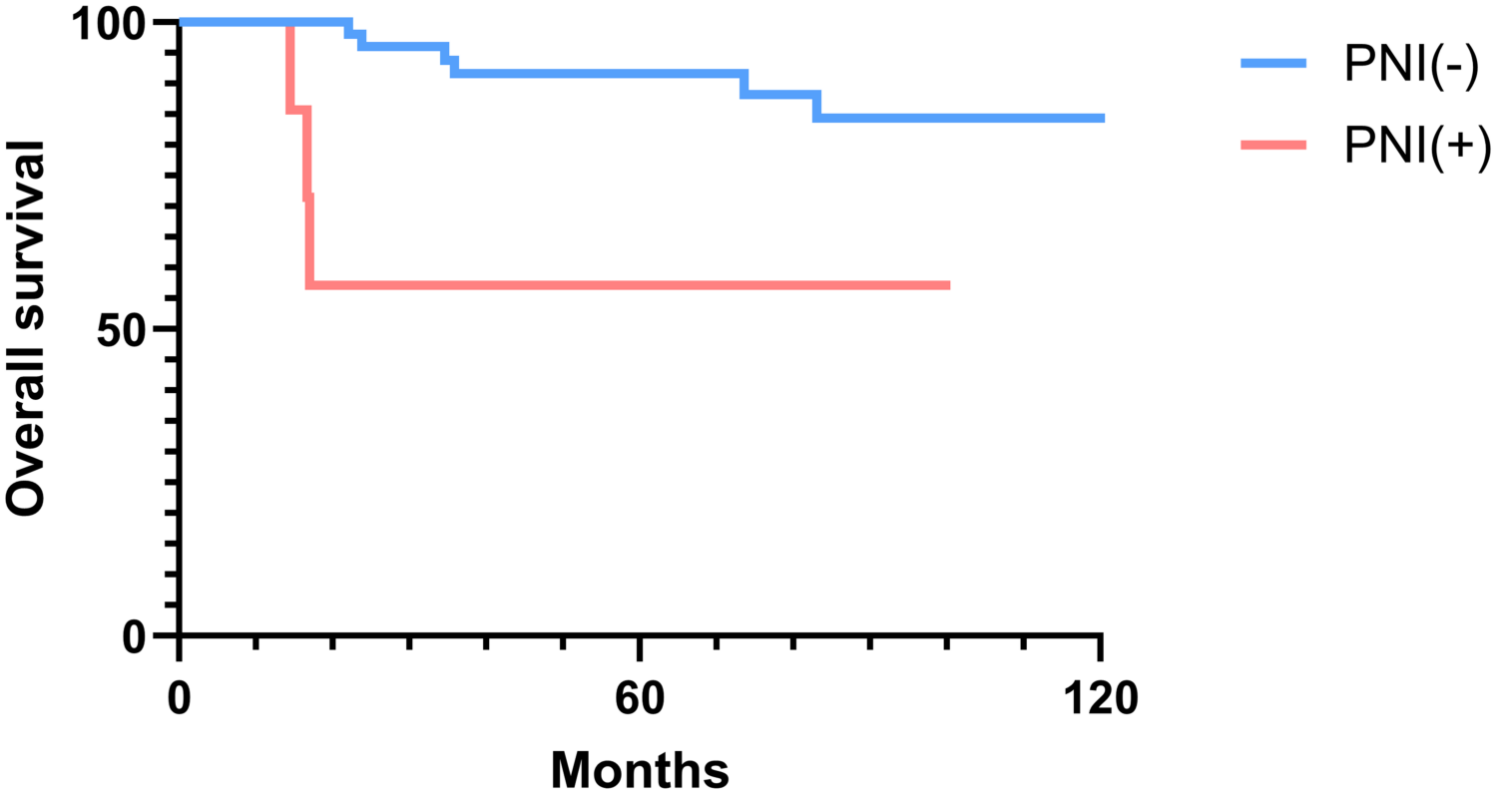
Overall survival curves showing significant differences according to perineural invasion (PNI).

**Figure 7:**
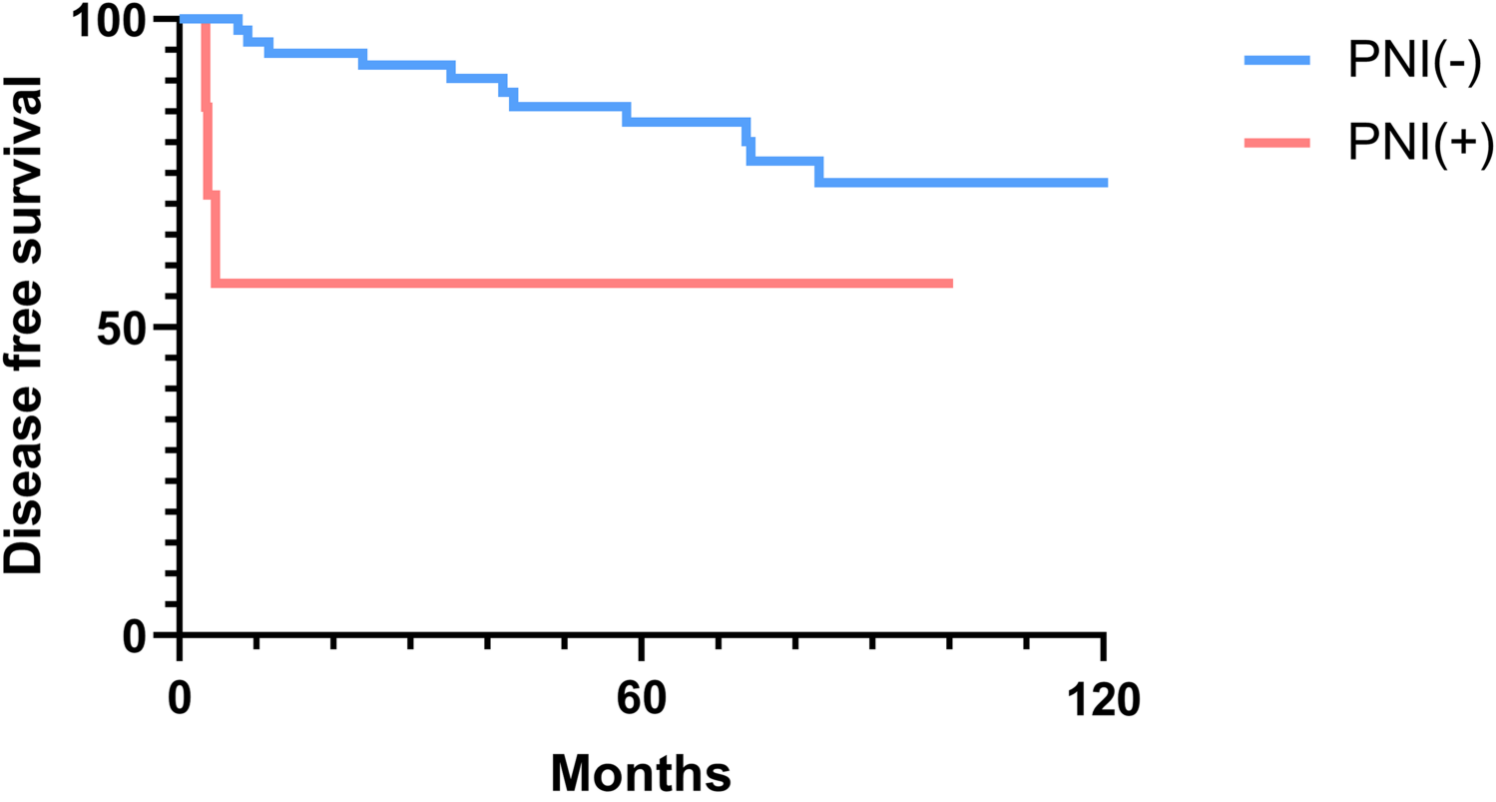
Disease-free survival curves showing significant differences according to perineural invasion (PNI).

The 2-, 5-, and 10-year OS rates were 91.7%, 88.0%, and 81.5%, respectively (Figure 1). The DFS rates at 2-, 5-, and 10-year were 88.9%, 81.0%, and 66.9%, respectively (Figure 1). The OS and DFS curves were significantly lower in patients with DOI > 5 mm (Figures 2, 3), LVI (Figures 4, 5), and PNI (Figures 6, 7).

## Discussion

This study analyzed the long-term treatment outcomes and the predictive factors for recurrence in 65 patients with pathologic Stage I/II OTSCC, treated surgically, with or without adjuvant therapy. To facilitate the development of more effective management strategies and improve patient outcomes, we also focused on identifying factors associated with early recurrence (within 6 months).

This study showed a near-equal gender distribution, with 52.3% male and 47.7% female patients, suggesting a nuanced shift from past evidence demonstrating higher incidences among males (2, 3). The mean age of 58.4 years is representative of the typical ages in other OTSCC studies, reinforcing that middle to older age groups are predominantly affected (2).

As shown in Figure 1, OS and DFS rates for Stage I/II OTSCC patients were high (in the ninety-percentile range at the 2-year mark and in the sixty to eighty-percentile range at the 10-year mark). These survival rates are consistent with or higher than those reported in previous studies (16). In a study of early-stage tongue cancer, Rusthoven et al. (16) reported the 5-year OS and cause-specific survival rates as 60.9% and 83.5%, respectively. The same study concluded that OTSCC was associated with poorer survival compared to other subsites of oral squamous cell carcinoma (16).

The standard treatment for OTSCC involves surgical resection, with or without adjuvant radiotherapy or chemoradiotherapy, unless the tumor is unresectable. The primary surgical approach for early-stage OTSCC traditionally involves wide tumor excision via transoral partial glossectomy or hemiglossectomy. Calabrese et al. (17) proposed an anatomically-based surgical approach for early-stage OTSCC, improving upon conventional wedge resections by enabling precise evaluation of deep surgical margins and visualization of the T-N tract, which is the soft tissue pathway between the primary tumor and cervical lymph nodes. Tagliabue et al. (18) demonstrated that the involvement of the T-N tract in advanced-stage tongue cancer was significantly associated with an increased risk of regional (nodal) and distant metastasis, as well as reduced survival. In the present study, among 13 patients who experienced recurrence, 12 exhibited locoregional recurrence, with one case specifically involving the T-N tract.

In addition to anatomical considerations, DOI remains an essential factor for determining the necessity of elective neck dissection (END). In this study, prophylactic SND was performed in most cases, except in cases with superficial T1 cancers, where the DOI was assessed as 1–2 mm based on preoperative imaging. Currently, a DOI of 3–4 mm is commonly used as a threshold for performing END to reduce the risk of occult metastasis (19, 20). However, sentinel lymph node biopsy (SLNB) has recently emerged as a valuable tool to further improve staging accuracy in early-stage OSCC. den Toom et al. (20) demonstrated that in cT1-2 OSCC patients, even with an optimal cutoff of 3.4 mm, occult metastases were still detected in 15% of cases below this threshold, indicating that DOI alone is an insufficient predictor of occult nodal metastasis. Additionally, den Toom et al. (21) showed that SLNB provides comparable accuracy to END in detecting occult nodal metastases for most oral cavity tumors, further supporting its role as an additional staging modality.

In this study, clinicopathologic factors significantly associated with recurrence included pathologic Stage II disease, LVI, PNI, and DOI >5 mm in the univariate analyses. As expected, T2 tumors showed a significantly poorer prognosis compared to T1 tumors. A DOI > 5 mm was associated with both increased recurrence (OR = 3.40, *p* = 0.04) and poorer survival (OR = 12.93, *p* = 0.02). The OR of PNI for recurrence and OS was 5.07 (*p* = 0.02) and 6.27 (*p* = 0.01), respectively. Moreover, the OR of LVI for recurrence and OS was 6.74 (*p* = 0.005) and 7.56 (*p* = 0.005), respectively. However, in the multivariate analysis, only LVI for recurrence and PNI for overall survival were confirmed as independent predictors (Tables 3, 4). Although our multivariate analysis did not verify all significant factors identified by univariate analysis, previous studies have shown associations of these prognostic factors with recurrence and survival outcomes in OTSCC patients (12, 13, 22). For example, a meta-analysis by Huang et al. (12) demonstrated that LVI was significantly associated with lymph node metastasis, especially in patients with early-stage OSCC, and that LVI correlated with poor prognosis. In the other study, PNI was found to be significantly associated with locoregional recurrence in early-stage OTSCC and with survival outcomes in all stages of OTSCC (13). Multiple pathological and biological features have been established as predictors of poor prognosis. Li et al. (22) comprehensively integrated these prognostic factors by proposing a histopathological risk model incorporating the worst pattern of invasion, PNI, and lymphocytic host response, which significantly predicts locoregional recurrence and disease-specific survival in low-stage oral squamous cell carcinoma. This model emphasizes the importance of identifying high-risk patients who are likely to fail primary surgical treatment alone and thus may require additional adjuvant therapy.

The surgical resection margin has gained recognition for its association with an increased risk of recurrence or poor survival. To date, what constitutes a safe surgical margin has not been clearly defined (23–25). In the literature, the definition of a safe surgical margin varies greatly, with 2 mm to 7 mm considered for the definition of a close margin by previous studies (25). Weijers et al. (23) compared local recurrences in patients treated for SCC of the tongue and floor of the mouth and found no statistically significant difference between patients with surgical margins > 5 mm and <5 mm. Likewise, a study by Nason et al. (24) showed that the survival outcomes and failure rates between 3 mm and 5 mm surgical margins were not significantly different. They also reported that both positive and close surgical margins of <3 mm were associated with poorer survival compared to surgical margins > 3 mm (24). In the study by Zanoni et al. (26), local recurrence-free survival was significantly associated with margins less than or equal to 2.2 mm.

In this study, we defined the safe margin as 2 mm. As shown in Table 2, the recurrence rate tends to be higher in cases with a surgical margin ≤ 2 mm (42.9%, 3 of 7) compared to cases with a surgical margin > 2 mm (17.2%, 10 of 58). However, no statistical significance was found between the two groups (*p* = 0.14). If we focus on local recurrence, in cases with ≤2 mm margin, local recurrence occurred in two (28.6%), whereas in the >2 mm group, local recurrence occurred in six (10.3%). Proportionally, local recurrence was also higher in the ≤2 mm group, although there was no statistical significance (*p* = 0.20). This study's lack of statistical significance might be due to the small number of subjects. Further research with a larger sample size is needed to determine what constitutes an adequately safe surgical margin.

Of all the OTSCC cases in this study, second and multiple primary tumors occurred in 10.8% of patients and were significantly associated with decreased OS in both univariate and multivariate analyses (Table 4). Our results concurred with the findings from Park et al. (27), which demonstrated 4.4% of second primary malignancy in early-stage OTSCC patients and salvage treatment success in 61.5% of patients. Similarly, Cai et al. (28) noted a poorer long-term prognosis for patients with multiple primary cancers compared to those with a single primary oral squamous cell carcinoma. Therefore, the development of secondary and multiple primary tumors represents a substantial risk factor for reduced survival, further complicating the management and prognosis of patients with OTSCC.

These findings underscore the importance of detecting and preventing second primary cancers as part of comprehensive care and follow-up strategies. Effective surveillance and intervention strategies, particularly for patients with known risk factors, are crucial for improving long-term outcomes. Among the 7 patients with second primary tumors, 5 were male, 3 were current smokers, and 5 reported alcohol consumption in this study. Modifying behavioral risk factors, such as smoking, alcohol consumption, and betel quid chewing, is critical for preventing second primary malignancies (29). However, 2 of the 7 patients were elderly females (over 70 years old) with no smoking or drinking history. In such cases, potential etiologies for second primary tumors might include chronic dental trauma, toxin exposure, or genetic factors (4, 30). Therefore, preventive measures addressing these risks are essential for patients.

In patients with oral tongue cancer, even early-stage disease, postoperative recurrences can occur within a short time. In this study, the mean time to recurrence was 40.7 months (median time to recurrence, 35.3 months), with the earliest recurrence detected at 3.4 months after surgery. Of the 13 patients with recurrence, 3 (23.1%) experienced early recurrence within 6 months post-surgery. All three patients with early recurrence presented with a T2 tumor, high DOI, and PNI. Early recurrence was significantly associated with PNI in the analysis using the Fisher's exact test. In contrast, the Cox proportional hazards regression model for early recurrences did not detect any significant factors related to early recurrence (Supplementary Table S3). This finding may be due to the limited number of patients with early recurrence in this study.

Further study to clarify the causes and related factors for early recurrence in patients with early OTSCC, including the molecular aspects of OTSCC, is warranted. Some studies have indicated that OTSCC is biologically distinct from other oral cancer subsites and is associated with the upregulation and downregulation of specific molecular markers, such as p16, p21, p53, Ki-67, VEGFs, and cyclin D1 (16, 31, 32). The identification of factors associated with recurrence, especially early recurrence, may contribute to the development of targeted treatment strategies and improved prognostic assessments for patients with early OTSCC.

Early recurrence *per se* may be indicative of a poor prognosis. In this study, all three patients with early recurrence ultimately died of the disease. Kernohan et al. (8) also showed that early recurrences at the primary site (<6 months after initial treatment) and late recurrences at the regional site (>6 months after initial treatment) were associated with worse outcomes.

This study has some limitations that should be addressed. First, the non-randomized retrospective study design is related to inevitable bias and limits the amount of information collected due to its dependency on retrieving data solely from charts. Second, the relatively small sample size may increase the risk of selection bias and limit the statistical power of the findings, potentially reducing their generalizability. Further studies with larger cohorts and additional investigations into risk factors, such as molecular markers, are warranted to better understand the underlying mechanisms of early recurrences and poor survival outcomes in early OTSCC.

In conclusion, univariate analyses identified LVI, PNI, DOI > 5 mm, and Stage II disease as factors significantly associated with recurrence and decreased survival. However, multivariate analysis verified only LVI as an independent predictor of recurrence, and recurrence, second primary cancers, and PNI as independent predictors of poor survival. To improve overall survival in patients with early pathologic Stage I/II OTSCC, the prevention and proper treatment of second primary tumors and recurrence are critical.

## Data Availability

The original contributions presented in the study are included in the article/Supplementary Material, further inquiries can be directed to the corresponding author.
