# Supplementary material for: Long-term oncologic outcomes and prognostic factors related to recurrences in pathologic Stage I/II early oral tongue cancer
Source: Front Surg. 2025 May 21;12:1534274. doi: 10.3389/fsurg.2025.1534274 (PMC12133764; doi:10.3389/fsurg.2025.1534274)
Supplement: Supplementary file 1 [file Datasheet1.docx]

Supplementary Material

# Supplementary Tables

Supplemental Table 1. Salvage treatment of patients with recurrences

| **Cases** | **Primary treatment** | **Recurrence site** | **Time to recurrence (months)** | **Salvage treatment** | **Prognosis** |
| --- | --- | --- | --- | --- | --- |
| 1 | Partial glossectomy, Rt | Tongue, base of tongue, hypopharynx, retropharyngeal LN | 7.6 | Palliative chemotherapy | Death with disease |
| 2 | Partial glossectomy, Lt with SND (I-III) | Level Ib, Lt. | 8.9 | SND, Lt (I, IV) with CCRT | Alive |
| 3 | Hemiglossectomy, Lt with SND (I-III) | Level I, II, V Lt | 11.6 | MRND and CCRT | Death with disease |
| 4 | Partial glossectomy, Lt with SND (I-IV) | Tongue | 43.4 | Wide excision of tongue cancer | Alive |
| 5 | Partial glossectomy, Lt with SND (I-III) | Tongue | 102.4 | Partial glossectomy, Lt. with SND (I-IV), Rt. | Alive |
| 6 | Partial glossectomy, Rt with SND (I-III) | Tongue | 133.4 | Wide excision of tongue cancer | Alive |
| 7 | Partial glossectomy, Lt with SND (I-III) | Tongue, Level I, III Rt. Level I, III, IV Lt. | 3.4 | Near-total glossectomy/ MRND, both / CCRT | Death with disease |
| 8 | Partial glossectomy, Lt with SND (I-III) | Tongue, SCM and Strap muscle, LN at level I-V | 3.7 | Palliative CCRT | Death with disease |
| 9 | Partial glossectomy, Lt + SND (I-III) / RT | Level Ib, Rt. | 4.7 | None (refused) | Death with disease |
| 10 | Hemiglossectomy, Rt + SND (I-III) / RT | Lung/liver/bone | 35.3 | Palliative chemotherapy | Death with disease |
| 11 | Partial glossectomy, Rt | Level II, Rt Neck muscle, obliteration of IJV | 42.0 | Palliative RT | Death with disease |
| 12 | Partial glossectomy, Rt with SND (I-III) | Tongue, Level I, Rt. | 58.1 | Partial glossectomy, with SND (level I) Rt. and RT | Alive |
| 13 | Hemiglossectomy, Lt with SND (I-III) | Tongue | 74.2 | Partial glossectomy | Alive |

Rt, right, Lt, left, SND, selective neck dissection, LN, lymph node, CCRT, concurrent chemoradiotherapy, RT, radiotherapy, MRND, modified radical neck dissection, SCM, sternocleidomastoid, IJV, internal jugular vein

Supplemental Table 2. Clinicopathologic factors related to early recurrence (within less than 6 months)

| **Factors** | | **Recurrence (-)**  **(N = 52)** | **Early Recurrence (+)**  **(N =3)** |
| --- | --- | --- | --- |
| Sex | M | 30 | 0 |
|  | F | 22 | 3 |
| Age | < 65 | 34 | 2 |
|  | ≥ 65 | 18 | 1 |
| Smoking | Non-smoker | 28 | 2 |
|  | Smoker | 24 | 1 |
| Alcohol | Non-drinker | 30 | 2 |
|  | Drinker | 22 | 1 |
| Stage | I | 31 | 0 |
|  | II | 21 | 3 |
| Histologic grade | Well | 32 | 1 |
|  | Moderately/poorly | 17 | 2 |
| LVI | No | 49 | 2 |
|  | Yes | 2 | 1 |
| PNI | No | 47 | 0 |
|  | Yes | 4 | 3 |
| DOI | ≤ 5 mm | 33 | 0 |
|  | > 5 mm | 19 | 3 |
| Surgical margin | > 2 mm | 48 | 2 |
|  | ≤ 2 mm | 4 | 1 |
| Adjuvant treatment | No | 46 | 2 |
|  | Yes | 6 | 1 |

DOI, Depth of invasion, LVI, lymphovascular invasion, PNI, perineural invasion

Supplemental Table 3. Cox proportional hazards regression model for early recurrences

| **Variables** | **Univariate** | |
| --- | --- | --- |
|  | **HR (95% CI)** | **p-value** |
| Sex (Female) | 82.40 (0.007 – 9.71×10^5^) | 0.36 |
| Age (≥65) | 0.91 (0.08 – 10.06) | 0.94 |
| Smoking | 0.60 (0.06 – 6.65) | 0.68 |
| Alcohol drinking | 0.68 (0.06 – 7.54) | 0.76 |
| Stage (II) | 90.51 (0.007 – 1.16×10^6^) | 0.35 |
| Histologic grade (moderately/poorly) | 3.63 (0.33 – 40.09) | 0.29 |
| LVI | 10.93 (0.98 – 121.68) | 0.05 |
| PNI | 4.99×10^4^(<0.01 – 5.11×10^43^) | 0.81 |
| DOI >5mm | 111.84 (0.007 – 1.87×10^6^) | 0.34 |
| Surgical margin (≤2mm) | 5.72 (0.52 – 63.19) | 0.16 |
| LVI, PNI, DOI>5mm | 100.17 (0.007 – 1.44×10^6^) | 0.35 |

DOI, Depth of invasion, LVI, lymphovascular invasion, PNI, perineural invasion

Supplemental Table 4. Clinicopathologic factors related to early recurrence vs. late recurrence

| **Factors** | | **Early Recurrence**  **(N = 3)** | **Late Recurrence**  **(N =10)** |
| --- | --- | --- | --- |
| Sex | M | 0 | 4 |
|  | F | 3 | 6 |
| Age (year) | < 65 | 2 | 7 |
|  | ≥ 65 | 1 | 3 |
| Smoking | Non-smoker | 2 | 7 |
|  | Smoker | 1 | 3 |
| Alcohol | Non-drinker | 2 | 6 |
|  | Drinker | 1 | 4 |
| Stage | I | 0 | 3 |
|  | II | 3 | 7 |
| Histologic grade | Well | 1 | 5 |
|  | Moderately/poorly | 2 | 4 |
| LVI | No | 2 | 7 |
|  | Yes | 1 | 2 |
| PNI | No | 0 | 9 |
|  | Yes | 3 | 0 |
| DOI | ≤ 5 mm | 0 | 4 |
|  | > 5 mm | 3 | 6 |
| Surgical margin | > 2 mm | 2 | 8 |
|  | ≤ 2 mm | 1 | 2 |
| Adjuvant treatment | No | 2 | 9 |
|  | Yes | 1 | 1 |

DOI, Depth of invasion, LVI, lymphovascular invasion, PNI, perineural invasion
